# New MT_2_ Melatonin Receptor-Selective Ligands: Agonists and Partial Agonists

**DOI:** 10.3390/ijms18071347

**Published:** 2017-06-23

**Authors:** Jean A. Boutin, Anne Bonnaud, Chantal Brasseur, Olivier Bruno, Nolwenn Lepretre, Peter Oosting, Sophie Coumailleau, Philippe Delagrange, Olivier Nosjean, Céline Legros

**Affiliations:** 1 Pôle d’Expertise Biotechnologie, Chimie, Biologie, Institut de Recherches SERVIER, 78290 Croissy-sur-Seine, France; anne.bonnaud@servier.com (A.B.); chantal.brasseur@servier.com (C.B.); olivier.bruno@servier.com (O.B.); sophie.coumailleau@servier.com (S.C.); olivier.nosjean@servier.com (O.N.); celine.legros@servier.com (C.L.); 2 Pôle d’Expertise Recherches & BioPharmacie, Institut de Recherches Internationales SERVIER, 92150 Suresnes, France; 3 DIVERCHIM S.A., 95700 Roissy-en-France, France; nolwenn.lepretre@diverchim.com (N.L.); peter.oosting@diverchim.com (P.O.); 4 Pôle d’Innovations Thérapeutiques en Neurosciences, Institut de Recherches, SERVIER, 78290 Croissy-sur-Seine, France; philippe.delagrange@servier.com

**Keywords:** molecular pharmacology, melatonin receptors, agonist, partial agonist, functional assays

## Abstract

The search for melatonin receptor agonists and antagonists specific towards one of the receptor subtypes will extend our understanding of the role of this system in relaying circadian information to the body. A series of compounds derived from a hit compound discovered in a screening process led to powerful agonists specific for one of the isoform of the melatonin receptor namely, MT_2_. The compounds are based on a poorly explored skeleton in the molecular pharmacology of melatonin. By changing the steric hindrance of one substituent (i.e., from a hydrogen atom to a tributylstannyl group), we identified a possible partial agonist that could lead to antagonist analogues. The functionalities of these compounds were measured with a series of assays, including the binding of GTPγS, the inhibition of the cyclic AMP production, the β-arrestin recruitment, and the cell shape changes as determined by cellular dielectric spectroscopy (CellKey^®^). The variations between the compounds are discussed.

## 1. Introduction

The neurohormone melatonin was discovered as *N*-acetyl-5-methoxytryptamine more than fifty years ago by Lerner and coworkers [1]. Its biosynthetic route follows a circadian rhythm, and is mainly performed by the pineal gland [2]. Other regions of the central nervous system and other tissues and cells such as the retina [3], skin, bone marrow, lymphocytes [4], and gastrointestinal tract [5] are capable of melatonin synthesis. The main acitivities of melatonin are mediated by two receptors (named MT_1_ and MT_2_) and a cytosolic protein (quinone reductase 2).

Melatonin plays a major role in almost all physiological processes, including modulation of hormonal secretions [6], regulation of cardiovascular functions [7], pain perception [8], depression [9], and immune system and core body temperature control [10,11]. Melatonin is a key participant in several pathological processes (see Boutin for recent review [12]) such as sleep disturbances and insomnia [13], cancer and inflammation [14], neurodegenerative diseases, diabetes, depression and anxiety [15,16,17]. Some cases of specificities were reported in which one particular isoform was implicated in a given pathology. In particular, several reports point to the possible key role of the receptor isotype MT_2_ in sleep [18] and in neuropathic pain [19], after initially referring to its implication in depression in mice [20]. Despite these reported numerous actions, ramelteon, agomelatine, and tasimelteon constitute the only melatonin analogues commercialized up to now [21,22,23] together with circadin, a melatonin galenic alternative form [24].

Although the endogenous role(s) and mechanism(s) of action of melatonin have not yet been fully elucidated, its therapeutic potential appears to be mediated via the activation of its two receptors (MT_1_ and MT_2_) that belong to the G protein-coupled receptors superfamily (GPCRs), although the inhibition of the enzyme quinone reductase 2 (previously known as *MT*_3_) might also have some role in the numerous therapeutic effects of melatonin [12]. Numerous studies of the two recombinant receptors from several species (human, sheep, rat or mouse) have revealed only minor differences between species. Particularly intriguing is that only a few agonist ligands with selective specificity towards the receptor subtypes have been found, and even fewer ligands with antagonistic functionality. Therefore, a better understanding of the melatoninergic system could lead to the discovery of new compounds, particularly potent ligands that are selective for the receptor subtypes [25,26,27]. A large number of high affinity non-selective ligands are described in the literature, among which ligands with clear receptor subtype selectivity, especially for MT_1_, remain elusive, despite a series of dimeric compounds claimed to be specific of this isotype (see Zlotos et al. [28]). To the contrary, MT_2_ specific ligands have been discovered and described more frequently.

**Figure 1:**
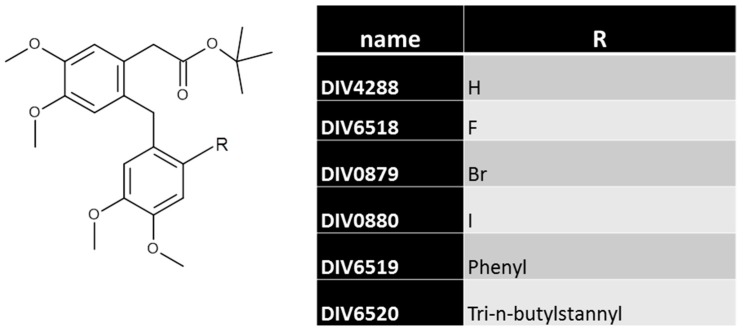
Structures of the compounds.

In the present work, we sought to prepare a series of putative specific ligands for the MT_2_ melatonin receptor, based on a hit compound (DIV0879) from our high throughput screening (HTS) campaigns [29]. Herein, we describe the analogues of DIV0880, a compound that we have used as a selective ligand in previous studies [30,31]. We attempted to characterize this compound and its derivatives as possible selective ligands for MT_2_ by observing their effects on the main protein Gi-mediated signaling pathways of melatonin receptors. We show that whatever the size of the R-substituent of the biphenyl moiety (see Figure 1), the compounds remain, at best, only partial agonists specific for the MT_2_ receptor while the original compounds (DIV0879 and DIV0880) were full agonists [30,31], a feature confirmed in the present work. However, as the size/hindrance of the substituents decreases, the agonistic partiality also decreases, rendering the compounds less and less effective as agonists.

## 2. Results

For the syntheses of these new compounds based on the biphenylmethane moiety, we questioned if the size of the substituent would lead to enhanced antagonistic activity specific toward the MT_2_ melatonin receptor. In the early phase of our designs, we did not anticipate that the compounds could be used in vivo at a later stage. The basic structures of the compounds were based on an initial result (from a random HTS). The model compound lacked the moiety frequently found in melatonin ligands (i.e., CH_2_CH_2_NHCOR), which was shown for agonists at this receptor by Mor et al. [32]. Another interesting feature of this molecule was its specificity toward the MT_2_ receptor, with no measurable affinity for the MT_1_ receptor. Although this feature was not unusual in this domain, it is worth pointing out that in this small series of compounds, none of the analogues regained affinity for the other subtype.

We decided to introduce more and more structural hindrance into the region of the molecule bearing a bromide (see Figure 1 for structures). We simply added larger and larger substituents at this position: from H, F, Br, I, Phenyl, to tri-n-butylstannyl [Sn(But)_3_]. We prepared these analogues without regard for the physical properties of the various substituents.

We first determined the binding affinities of those compounds that bound to the MT_2_ receptor, as evaluated by a binding assay using 2-[^125^I]-iodo-melatonin as a ligand (Table 1). All the compounds, interestingly, presented binding affinities in the nanomolar range, with the exception of the compound with the largest substituent, the Sn(But)_3_ derivative, which displayed a binding affinity for MT_2_ that was lower by a factor of a log value. As pointed out previously, all compounds remained MT_2_-specific, as binding at MT_1_ was recorded in the low micromolar range, presenting, therefore, a difference of 3 log values between the two types of receptors.

**Figure 2:**
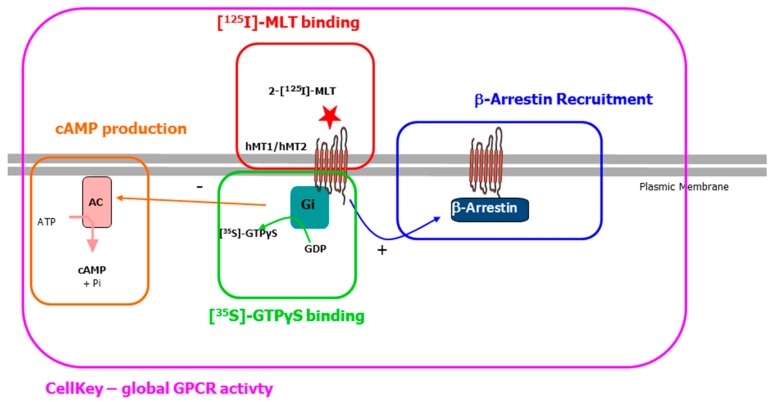
Schematic representation of the melatonin receptor pathways worked out in this paper. **Red**: the binding of the radio ligand 2-[^125^I]-melatonin onto the receptor gives an affinity of the receptor for the compound; **Green**: The binding of the nonhydrolysable, radioactive GTP analogue [^35^S]GTPγS is a measure of the capacity of the ligand molecule to recruit the induced the Gi protein, that leads to the Gi-mediated inhibition of the adenylate cyclase; **Orange**: The measure of the cyclic AMP (cAMP) production by adenylate cyclase is a finer image of the capacity of the ligand at the receptor to recruit Gi and to inhibit this production; **Blue**: Ligands that are agonist tends to provoke the recruitment of β-arrestin, as a first step towards the desensitization of the receptor; **Purple**: Cellular dielectric spectroscopy (Cellkey^®^) is a technology capable of detecting whole-cell response to a treatment—such as the binding of an agonist at a receptor by measuring modifications in electric impedance in a dose-dependent manner.

A sketch of the signaling pathways visited in the present work is provided in Figure 2. Because affinity does not presume the functional nature of the compounds (whether agonist or antagonist), we turned to several functional assays: GTPγS is a test that is dependent on the activation of the Gi pathway [33]. As can be seen from Table 2, the compounds activated this particular pathway up to the iodine-bearing compound, DIV0880, but larger derivatives did not, as the presence of even larger substituents (phenyl or Sn(But)_3_ impaired the respective compound ability to activate this particular Gi-dependent pathway. Furthermore, while they all acted as agonists (i.e., being able to activate the pathway by themselves, without melatonin), they were only partial, poorly effective agonists, as their potencies were in the 30% range of the melatonin potency with respect to the same receptor, within the same pathway.

The [^35^S]-GTPγS binding assay measures directly the activation of Gi. This activation can—but does not always—lead to the inhibition of the cAMP production, once the adenylate cyclase is induced by forskolin. To better evaluate this parameter, with the goal to characterize the compounds as agonists at the MT_2_ receptor, we chose to measure the variations of the cAMP production as it can be induced by the various agonists (Table 3). The first four compounds activated the Gi-dependent pathway with potencies similar to those obtained while measuring the [^35^S]-GTPγS binding pathway. EC_50_ is defined as the half maximal effective concentration. It is sometimes expressed as its Log, pEC_50_. For the last two larger compounds, however, if activation of the pathway occurred, they were with pEC_50_ values that remained poorer by 2 log units than the first compounds. The Emax is the maximum possible effect for the agonist. Interestingly, the E_max_ values of the compounds were all close to 100%, the score of the melatonin itself. This discrepancy is due to the nature of the assay. Indeed, the GTPγS binding assay is a static one, limited roughly by the number of binding sites, and the cAMP assay is an enzymatic one, the only limit of which is the time during which the enzyme produces cAMP. In other words, the result at a given time, for the cAMP assay, is similar from an agonist to another, while the pEC_50_ will be dependent on the potency of the molecule. It is therefore slightly wrong to use cAMP as a potency assay. It should be limited to an “affinity measurement” one. This outcome strongly suggests that the compounds were full agonists of this pathway. Interestingly, when one compares the values obtained with this cAMP production assay with the previous test, one finds the higher sensitivity of the latter to be key. Indeed, the two larger compounds seemed to be completely unable to activate the Gi pathway, while the cAMP production, a more dynamic assay, reveals they can.

To summarize, the compounds of identical chemical structure but differing in the size of a single substituent (from the hindrance point of view) could activate, as agonists, the Gi pathway. Despite their differences in potencies, they remained strong agonists toward only the MT_2_ receptor. As is often the case with the melatonin receptors, the complexity of the pathways makes it difficult to find pure agonists or antagonists, even if they are not specific of one of the subtypes. In the present work, we identified strong binders, specific for MT_2_ but, for some of them, they were only partially effective, depending on the signaling pathway measured.

The investigation of those compounds continued with determination of the β-arrestin recruitment. This test evaluates the agonist capacity of the compound. Indeed, viewed in a simple way, the compounds that are agonists lead to the desensitization of the pathway. For desensitization to occur, the receptor must be internalized. The first step of the internalization is the recruitment of the β-arrestin (see Claing et al. [34], and Reiter and Lefkovitz [35], for further details). This desensitization process is used by the vast majority of 7TMRs (see reviews and references thereof: Pitcher et al. [36], and Lefkowitz and Shenoy [37]).

The data were obtained using CHO-K1 cells expressing either hMT_1_ or hMT_2_ receptors. Results are given as the mean ± S.E.M. for at least three experiments.

We developed a complementary assay to measure the recruitment of β-arrestin as a function of the concentration of the compound. Because the test is built with brand new cell lines using enzyme fragment complementation assay Technology (PathHunter^®^, DiscovereX) [38], the pharmacology of these cell lines should be validated and the findings with reference compounds double checked with standard methodology. This process has been done and confirms the similarity of the cell lines, as well as the robustness of the assay (Dupré and Boutin, in preparation). Only agonists can elicit some recruitment in this model, although some agonists do not, while antagonists never do. As indicated in Table 4, despite the low affinity of the compounds for MT_1_ (in the micromolar range), they elicited this recruitment. This is a phenomenon often seen with complementary assays: the affinity of the two parts of the enzyme for each other tends to drive, at least in part, the agonist-induced pathway. The efficacy of these compounds, compared to melatonin, was partial, in the 60% range. Furthermore, these pEC_50_ values are of the same range as the affinity of the compounds for the MT_1_ receptor. In other words, the test is clearly very sensitive and can be used to evaluate poor affinity phenomena. To the contrary, for the MT_2_ receptor, the same compounds were able to recruit β-arrestin in the same manner as for melatonin itself (namely, 100%), and even in some cases, beyond that (140%), despite a slightly diminished pEC_50_ in this test. The same ability applies for the two most hindered compounds that were ineffective, partial agonists in this test.

**Figure 3:**
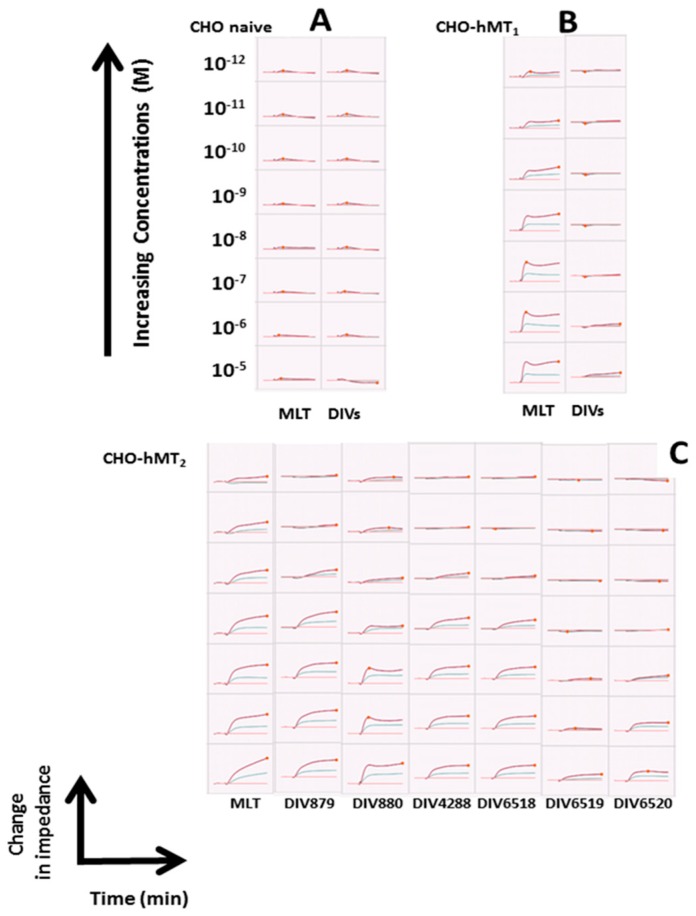
Screen capture of the CellKey^®^ experiments. Within a square, the changes in impedance translate as a function of time (20 min total) and as a function of concentration of ligands (within a column, from the top line (1 × 10^−12^ M) to the bottom line (1 × 10^−5^ M) are monitored, and the inflection point could be used to construct graphs, leading to half maximal inhibitory concentration (IC_50_) calculations. Note the absence of response in naïve cells (**A**), the response of melatonin (MLT) only in MT_1_-expressing cells (**B**) and the various curves for the MT_2_-specific ligands (**C**). These captures are examples taken from a series of 2–5 experiments, depending on the compounds.

To further characterize these compounds, we tested them using the cellular assay, Cellkey^®^, described elsewhere [39] and applied to systems other than the melatoninergic receptors (e.g., δ opioid receptors [40], and lysophosphatidic acid receptor 1 [41]). This methodology is a label-free cellular technique that measures the changes in the conductance (dielectric impedance) of cells that occur when the cellular receptor binds a ligand, resulting in a change in the shape of the cell. Depending on the signaling pathway, it has been shown that the variation of this impedance curve as a function of time (and of concentration) is somewhat specific of one of the canonic GPCR signaling pathways (see Figure 3).

**Figure 4:**
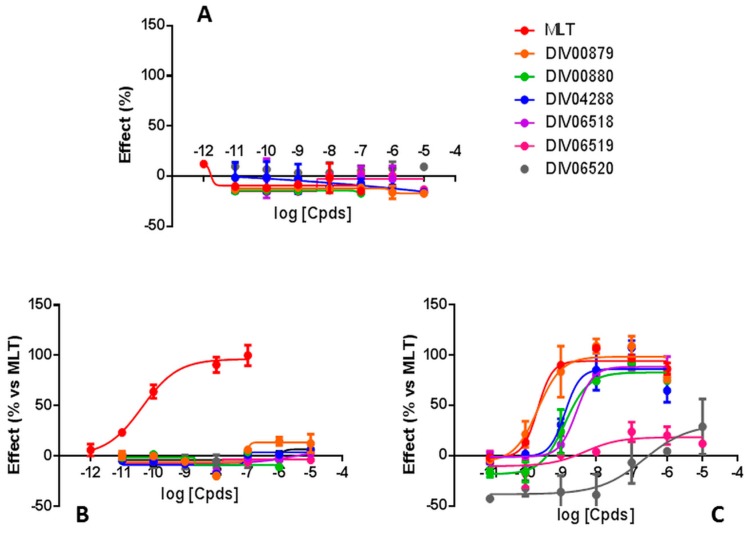
CellKey measurements melatonin receptors MT_1_ and MT_2_ response elicited by DIV0879 analogues. CHO cells transfected with MT_1_ (**A**) and MT_2_ (**B**) as well as naïve CHO cells (**C**) were treated with melatonin (MLT) and the analogues as indicated in the color key in the figure over the concentration range of 10^−12^ to 10^−5^ M. At each concentration, the change in electrical impedance was measured as described in the Experimental section. The data were corrected for baseline response and are normalized with respect to the response to melatonin at the same concentration. The data represent the mean ± SEM of five trials. SEM is standard error of the mean.

As the conformation of the cell is altered, its ability to conduct a current is also altered. The method has been previously assessed and applied to the melatoninergic system in our laboratory [42]. In this study, we have used cells that were transfected with the MT_1_ or MT_2_ receptors and compared the results to naïve CHO cells. Figure 4 compare the changes in electric impedance of each category of cells as a function of the concentration of the compounds. In control non-transfected cells, none of the compounds elicited a response, indicating that in the absence of melatonin receptors, there was a baseline level of specificity for endogenous receptors spontaneously expressed in the cell line (Figure 4A). For cells containing MT_1_ (Figure 4B), the compounds were not potent enough toward this receptor to elicit any signal, while melatonin itself did. It is interesting to notice that there are three kinds of compounds in this series, excepting melatonin. Indeed, melatonin clearly shows a capacity to recruit the β-arrestin on both receptors, as expected, and as a natural agonist should do (red trace, Figure 4B,C). The first compound is DIV0879 (orange trace, Figure 4C) that is very similar to melatonin in this test, as it recruits β-arrestin with identical potency and efficiency, when compared to the natural agonist. The next group comprises DIV4288 (blue trace, Figure 4C), DIV6518 (purple trace, Figure 4C) and DIV880 (green trace, Figure 4C). They all are less potent in recruiting β-arrestin, but remained capable of doing so with almost 100% potency, close to the natural agonist. The last group is formed of the two last compounds, DIV6519 and DIV6520 (pink and grey traces, Figure 4C), the most hindered ones, and they showed poor capacity to act as agonists at the MT_2_ receptor.

An overall comparison can be made at this stage between the various signaling pathways. The features of the β-arrestin recruitment results are also interesting when they are observed together with the data obtained first with the GTPγS assay. Indeed, the behavior of the two last compounds, DIV6519 and DIV6520 are similar and they do not result in the binding of GTPγS, as if they were acting like potent antagonists, according to the fact that their affinities for MT_2_ are 0.5 and 36 nM, respectively. When comparisons were made with the cAMP production assay, though, the situation was less clear. Indeed, both compounds, DIV6519 and DIV6520, could induce a production of cAMP, albeit with a poorer efficiency, compared to the other compounds of the series and melatonin itself, reinforcing the idea that they are poorly potent agonists to a point that they can behave as antagonists. The final point would be to comment on the Cell Key^®^ assay. Due to the relative newness, it is hard to know exactly what to expect. It is certain that melatonin, on both isoforms of the receptor, behaves roughly the same way, even though on MT_1_ receptor, a peak of respond appears at low concentrations, a feature that melatonin on MT_2_ does not induce. For the other compounds, the overall picture is the same: concentration-dependent enhanced response of impedance dielectric signal shape (red traces, bottom of Figure 3), with enhanced amplitude at high concentrations. The last two compounds (DIV6519 and DIV6520), behave differently, with a poor increase in the signal, if any.

## 3. Discussion

The search for ligands for the melatonin receptors has been a quest for many laboratories since the pioneering work on melatonin analogues by Taborsky and McIsaac [43], and Kobayashi et al. [44]. The compounds discovered over recent decades fall into four categories: (1) the bicyclic compounds, loose analogues of the indole cycle of melatonin; (2) the tricyclic compounds that are simply one more cycle fused to the bicyclic ones; (3) the unusual case of the unique and selective MT_1_ antagonist verapamil-derived D600 [45] that is everything but specific (it affects many other targets including the voltage-dependent calcium channels); and (4) the present compound(s) in which the two cycles (of the first category) have been separated by a methylene bridge (notably, the series described by Hu et al. [46] encompasses the same global scheme). Most of these compounds bear the acetylamidoethyl side chain.

A complete discussion of the melatonin receptor ligands can be found in the International Union of Basic and Clinical Pharmacology (IUPHAR) compendium [47], as well as in the review of Zlotos et al. [28] where DIV0880 is mentioned as a selective MT_2_ radioligand. The present series of compounds exemplifies further the series of diphenylmethylene-based compounds [46], described by Hu et al. in a study mainly dealing with phenoxyphenyl compounds [46]. These compounds differ from the current agonists in that they do not bear the canonic melatonin chain (i.e., acetylamidoethyl, simply referred to as “the amide moiety” [25]) that most of the agonists of melatonin receptors possess (see review by Spadoni et al. [25]).

Antagonists at melatonin receptor(s) have been difficult to obtain. Most of the compounds described thus far are partial agonists that behave completely differently—from a functional point of view—whether they are characterized by one or another of the numerous functional pathways downstream of the seven trans-membrane domain G-coupled receptors (Legros, C.; Boutin, J.A., in preparation).

At that stage, the notions of agonists, antagonists and partial agonists should be briefly discussed. Agonists, often natural compounds, are capable of eliciting a full activation of the receptor pathway in a functional assay. This is followed very rapidly by a desensitization process in which the receptor is internalized in the cell, in order to make it unavailable to further activation. This process is often targeted by the recruitment of β-arrestin. Antagonists are compounds that impair the agonist to bind (and to act) without having its own capacity to activate the receptor. Partial agonists are defined by their partial capacity to activate the receptor. The IUPHAR recommendations state that “…a partial agonist that when occupying all the receptors produces a maximal response that is half that of a full agonist (under the same experimental conditions), has an efficacy of unity. Efficacy is both agonist- and tissue-dependent” [48], and hence, less than 50%. To these notions, one can add the notion of ineffective partial agonists [49] that translate simply by a very poor activation of the receptor, being then different from an antagonist, since those ones are not able to activate the receptor while partial agonists do, albeit ineffectively. Unfortunately, none of those authors launched a mathematical limit between ineffective and effective partial agonists; neither did the IUPHAR ad hoc committees (M. Spedding, personal communication). We tried to respect these general ideas based on common sense in the present paper.

In multi-domain receptors that mediate multiple functions, the notion of biased agonism has been introduced. Biased agonists are ligands that selectively affect only one signaling pathway within a multifunctional receptor. We have described new and original compounds exhibiting partial agonism favoring the MT_2_ receptor functionality. While the concept of biased agonism is not new, as far as we know, it has never been applied to melatonin receptors.

We wanted to explore the behavior of simple analogues of the powerful original compound discovered from an HTS campaign, with a nanomolar affinity at MT_2_. Despite our goal to generate antagonists simply by enhancing the steric hindrance of a substituent at a given position, the compounds we prepared were partial to very partial agonists (from effective to ineffective agonists), which were still able to generate internalization of the receptor, a feature that antagonists do not possess. Most of the ligands described in the literature over the last three decades have been fused bicyclic compounds with various heteroatoms in the cycle(s), bearing the so-called “amide” moiety [25], and having a methoxy substituent on the aromatic rings. All of these specific features of melatonin ligands—particularly agonists—have been described with a large number of variations within these features and in their proximity (see Zlotos et al. [28] for a review of the literature).

The present series of compounds is slightly different in structure. First, rather than bearing the characteristic ethylacetamide moiety, these new derivatives have a shorter and more hindered CH_2_COO*t*Bu moiety. It seems unlikely that this substituent is a bioisostere of the CH_2_CH_2_NHCOR chain. The second structural difference is that the new compounds do not have a fused cyclic structure, but instead, a methylene chain separates the rings.

We were surprised to see large substituents such as Sn(But)_3_ and phenyl directing the corresponding compound toward the behavior of ineffective partial agonists (e.g., less than 20% of melatonin at MT_2_ receptor), remaining slightly agonistic for MT_2_. Although there are several models of the receptors that have been published, it has not been easy, so far, to elaborate on this structure–function relationship.

We hope to further explore this series of compounds by using various approaches that can analyze the melatonin receptor structural biology, e.g., the purification of a functional receptor [50,51] (albeit MT_1_ only, at the moment) and the models that have been described recently for the receptors [52,53]). Hopefully, the combination of new chemical series with strong affinity and clear functionality toward the melatonin receptor(s) will help assess new tools to improve our understanding of the role of melatonin within and outside its receptor routes.

## 4. Materials and Methods

### 4.1. Synthesis of the Compounds

The synthesis of compounds DIV0879 (the bromide derivative) and DIV0880 (the iodinated derivative) have been previously described [30]. All the compounds were purified and analyzed by mass spectrometry and nuclear magnetic resonance NMR. The crude graphs corresponding to the six compounds are added as supplementary files (Figures S1–S11).

#### 4.1.1. DIV6518

**Figure 5:**
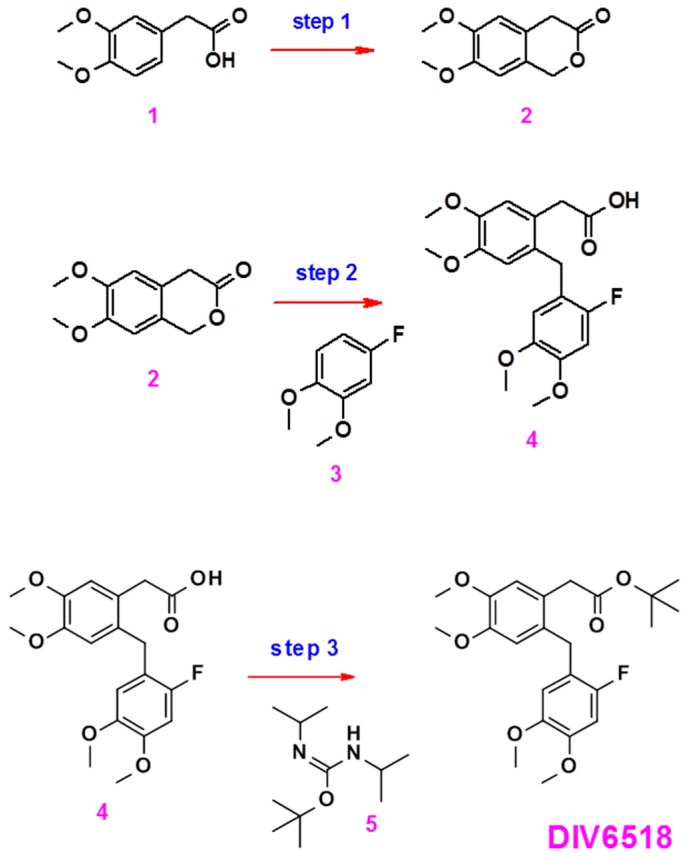
Schematic representation of the synthesis of DIV6518.

Compound DIV6518 was prepared in three steps according to the scheme (Figure 5).

##### Preparation of Compound **2**

Compound **1** (51.4 g) was mixed with a solution of acetic acid (150 mL) and concentrated hydrochloric acid (50 mL). Formalin (50 mL) was added and the mixture was heated at 90 °C for 2 h. The solution was allowed to cool to room temperature and poured into water (1.2 L). The aqueous layer was extracted three times with dichloromethane. The combined organic layers were washed successively with a saturated sodium bicarbonate solution (three times) and brine, dried over magnesium sulfate, filtered and concentrated to give a yellow solid. The crude solid was triturated in isopropyl ether to give the expected product: a light yellow solid (33.3 g, yield 61%).

##### Preparation of Compound **4**

Compound **2** (2.64 g) was dissolved in formic acid (26 mL) and compound **3** (Alfa Aesar, Haysham, UK, A17936): 4-Fluoro-1,2-dimethoxybenzene, 2 g) was added. The mixture was heated at 90 °C overnight. A thin layer chromatography TLC analysis (dichloromethane/ethyl acetate 8/2) indicated that compound **2** had been totally consumed. The mixture was allowed to cool to room temperature and poured into water. The aqueous phase was extracted three times with ethyl acetate. The combined organic phases were washed with water until the pH of the aqueous washings was neutral. The organic phase was finally washed with brine, dried over magnesium sulfate, filtered and concentrated to give a crude product, (4.57 g). The crude solid was triturated in isopropyl ether to give the expected product: brown solid (2.7 g, yield 58%).

##### Preparation of the Final Compound DIV6518

Compound **4** (2 g) and compound **5** (Diverchim, Roissy, France (DIV0439) 2-tert-Butyl-1,3-diisopropylisourea, 1.65 g) were dissolved in dichloromethane (25.5 mL). The mixture was stirred at room temperature overnight. An aliquot was analyzed by NMR showing the presence of residual starting material. An additional batch of compound **5** (1.65 g) was added and stirring was continued overnight. An aliquot was analyzed by NMR indicating complete conversion of starting material. Water (25 mL) was added and the biphasic mixture was stirred for 45 min. The solid was filtered and the filter cake was rinsed with dichloromethane. The filtrate was collected and the phases were separated and the aqueous phase was further extracted with dichloromethane. The combined organic phases were washed with brine, dried over magnesium sulfate, filtered and concentrated to give a semi solid compound (2.46 g). The resulting crude solid was purified twice by column chromatography (Silica gel, heptane/ethyl acetate 3/1 and dichloromethane/diisopropyl ether 98/2) to give the expected product: white solid (270 mg, yield 11%).

#### 4.1.2. DIV4288

**Figure 6:**
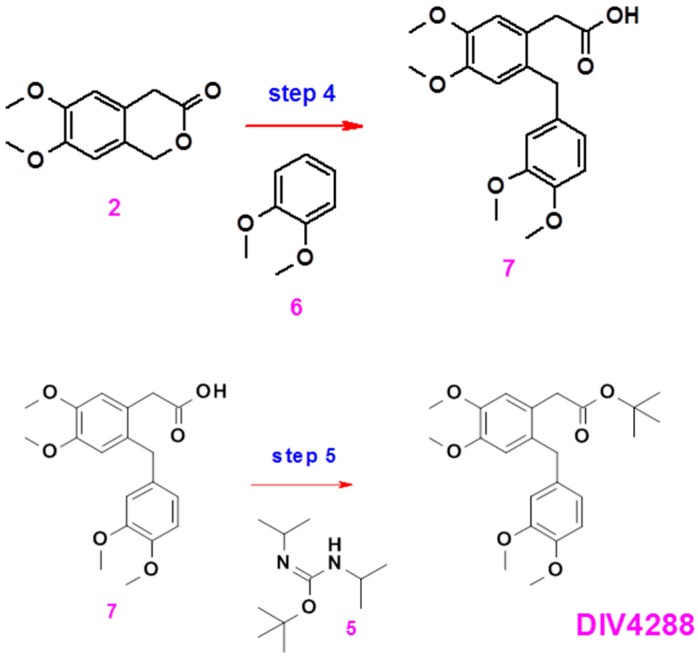
Schematic representation of the synthesis of DIV4288.

Compound DIV4288 was prepared in two steps from intermediate **2** according to the synthesis scheme (Figure 6).

##### Preparation of Compound **7**

Compound **2** (12.1 g) was dissolved in formic acid (121 mL) and compound **6** (8.9 mL) was added. The mixture was heated at 90 °C for 4 h. An aliquot was analyzed by NMR, showing complete consumption of the starting materials. The mixture was allowed to cool to room temperature and poured into water. The aqueous phase was extracted four times with ethyl acetate. The combined organic phases were washed with brine, dried over magnesium sulfate, filtered and concentrated to give a brown oil (29 g, 178-080-B). The crude oil was purified by column chromatography (Silica gel, dichloromethane/ethyl acetate 9/1) to give the expected product: brown solid (2 g, yield 10%).

##### Preparation of Compound DIV4288

Compound **7** (2 g) and compound **5** (Diverchim, Roissy, France (DIV0439) 2-tert-Butyl-1,3-diisopropylisourea, 3.71 g) were dissolved in dichloromethane (30 mL). The mixture was stirred at room temperature for 5 h. A TLC analysis (dichloromethane/methanol 95/5) showed the presence of residual starting material. An additional batch of compound **5** (1.86 g) was added and stirring was continued overnight. An aliquot was analyzed by HPLC indicating virtually complete conversion of starting material. The suspension was filtered and the filtrate was concentrated. The residue was triturated in diethyl ether (1 V) and the filtrate from this operation was concentrated to give a brown oil (3.9 g, 178-092-Ef). The resulting crude solid was combined with a previously obtained batch and purified by column chromatography (Silica gel, dichloromethane) to give a brown semi solid that was triturated in pentane to result in the expected product: white solid (2.5 g, yield 42%).

#### 4.1.3. DIV6519

**Figure 7:**
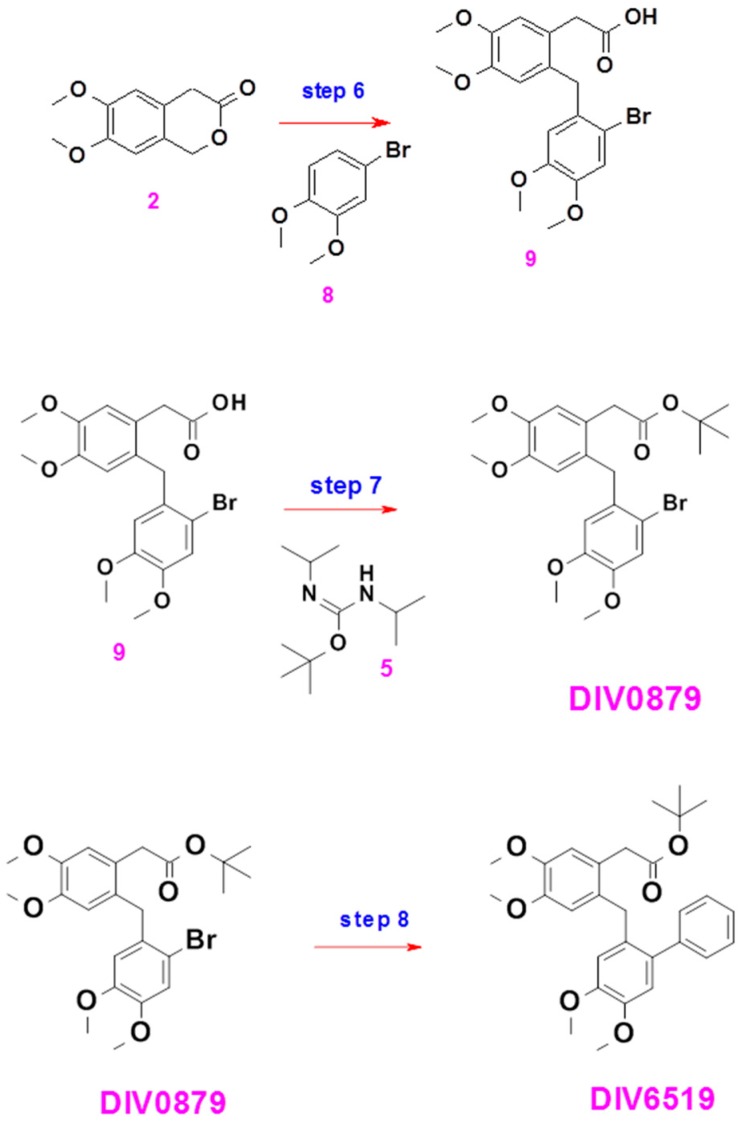
Schematic representation of the synthesis of DIV6519.

Compound DIV6519 was prepared in three steps from intermediate **2** according to the synthesis scheme (Figure 7).

##### Preparation of Compound **9**

Compound **2** (5 g) was dissolved in formic acid (25 mL) and compound **8** (Acros, Geel, Belgium (107,540,250, 6 g) was added. The mixture was heated at 100 °C for 3 h and for 7 h overnight. The mixture was poured into water and extracted three times with ethyl acetate. The combined organic phases were washed with brine, dried over magnesium sulfate, filtered and concentrated to give a brown semi solid. The crude product was triturated in a mixture of diisopropyl ether/ethyl acetate 7/3 to give a brown solid. The solid was crystalized from toluene to give the expected product: brown solid (6 g, yield 59%). A second crop of product was obtained by trituration of the different mother liquors in a mixture of heptane/ethyl acetate 1/1 to give a brown solid (1.05 g, yield 10%).

##### Preparation of DIV0879

Compound **9** (1.04 g) and compound **5** (1.58 g) were dissolved in dichloromethane (30 mL). The mixture was stirred at room temperature overnight. A TLC analysis (dichloromethane/methanol 8/2) showed the presence of residual starting material. An additional batch of compound **5** (0.79 g) was added and stirring was continued overnight. The reaction mixture was concentrated to dryness to give a crude product. The product was purified by column chromatography (Silica gel, dichloromethane/ethyl acetate 85/15) to give a product that was not pure (320-006-C1). The product was triturated in a mixture of pentane/ether. Concentration of the filtrate led to a product that was further purified by column chromatography (Silica gel, gradient heptane/ethyl acetate) to give the expected product, DIV0879 as an off-white solid (749 mg, yield 63%).

##### Preparation of Compound DIV6519

DIV0879 (199 mg, 0.41 mmol), sodium carbonate (123 mg, 1.16 mmol) and phenyl boronic acid (86 mg, 0.71 mmol) were dissolved in the ethanol/water mixture (5 mL). The mixture was placed under nitrogen atmosphere and palladium tetrakis triphenylphosphine (25 mg, 0.02 mmol) was added. The reaction was heated at 85 °C overnight. A TLC analysis (dichloromethane/methanol 99/1) indicated that all the starting materials had been consumed. The mixture was concentrated to dryness to give a crude product (600 mg, 210-164-B). The product was purified by column chromatography (Silica gel, dichloromethane/methanol 99/1) resulting in a colorless oil that solidified upon standing (200 mg, 210-164-C1). The product was triturated in pentane (15 mL) to give the expected compound: white solid (90 mg, yield 46%).

#### 4.1.4. DIV6520

**Figure 8:**
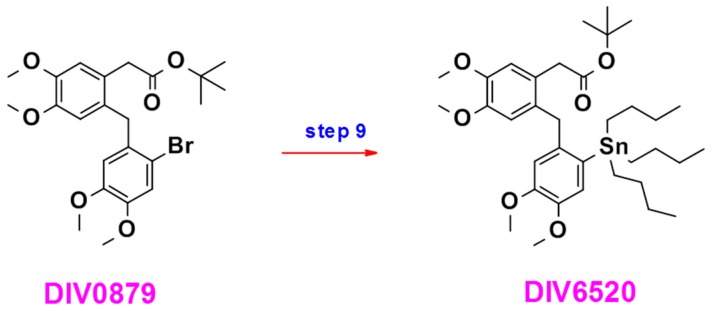
Schematic representation of the synthesis of DIV6520.

Compound DIV6520 was prepared in one step from intermediate **10** according to the synthesis scheme (Figure 8).

##### Preparation of Compound DIV6520

DIV0879 (200 mg, 0.41 mmol) and hexabutyl tin (603 mg, 1.04 mmol) were dissolved in the ethanol/water mixture (5 mL). The mixture was placed under nitrogen atmosphere and palladium tetrakis triphenylphosphine (25 mg, 0.02 mmol) was added. The reaction was heated at 100 °C overnight. A TLC analysis (toluene/ethyl acetate 9/1) indicated that virtually all the starting materials had been consumed. The mixture was allowed to cool to room temperature and was poured into a solution of potassium fluoride (20 mL). The suspension was filtered over Celite and the filtrate was extracted with ethyl acetate. The organic phase was dried over magnesium sulfate, filtered and concentrated to give a brown paste (0.73 g). The product was purified by column chromatography (Silica gel, gradient toluene/ethyl acetate) resulting in colorless oil that was further purified by preparative HPLC to give the expected compound: light yellow oil (60 mg, yield 22%).

### 4.2. Biological Experiments

#### 4.2.1. Cell Line and Membrane Preparation

We described these methodologies previously and used them as we described [30].

#### 4.2.2. 2-[^125^I]-Iodomelatonin Binding Assay

We used these assays exactly as we described previously [54].

#### 4.2.3. Homogeneous Time Resolved Fluorescence HTRF cAMP Assay

For the cellular cAMP production measurement, we used the assay we validated and described previously [30].

#### 4.2.4. [^35^S]-GTPγS Binding Assays

The [^35^S]-GTPγS binding assay we used has been thoroughly described previously by us on other systems [33] or on melatonin receptors [30].

#### 4.2.5. β-Arrestin Recruitment Assay

The β-Arrestin recruitment was monitored using PathHunter CHO-K_1_-MT1_2_R β-arrestin cell line from DiscoveRx^®^ (Birmingham, UK), essentially as described by Sakurai et al. [55] and recently partly assessed by Gdahou et al. [56]. Cells were cultured at 37 °C, 5% CO_2_ in F12-GlutaMAX medium (Thermo Fisher Scientific, Waltham, MA, USA) supplemented with 10% heat inactivated fetal bovine serum. Engineered MT_1_-2R and β-arrestin expression was maintained using antibiotic selection (Hygromycin B 300 μg/mL, Geneticin 800 μg/mL). For assays, cells were washed in Dulbecco’s Phosphate-Buffered Saline (DPBS), resuspended using Cell Dissociation Solution (Sigma-Aldrich, St. Louis, MO, USA) and seeded into CELLSTAR 384-well microplates (Greiner Bio-one) at 6500 cells per well in 20 μL PathHunter Cell Plating Reagent, then incubated at 37 °C, 5% CO_2_ for 24 h. After compound incubation (in 1.5% final concentration dimethyl sulfoxide (DMSO) for a final volume of 25 μL) for 90 min at 37 °C, 5% CO_2_, PathHunter detection reagent was added and the enzyme fragment complementation reaction was carried out for 120 min with shaking at room temperature. Chemiluminescent signal was measured on a MicroBeta TriLux (PerkinElmer, Villebon-sur-Yvette, France). This approach has been further validated and characterized and will be published in a forthcoming paper [57].

#### 4.2.6. Cellular Dielectric Spectroscopy

The impedance measurement assay uses CHO-K1 cells expressing human MT_1_ and MT_2_ receptors. We previously described this approach in our work on sheep melatonin receptor cloning [58]. This test was also previously assessed, and used in our group with the human receptors [42]. It was used here with minor modifications: the cells were plated (40,000 cells per well) onto MDS Analytical 96-well assay plates with embedded electrodes (MDS Analytical, Concord, ON, Canada). The plates were incubated at 37 °C, CO_2_ 6% for 48 h. Prior to the measurement, cells were washed three times with Hanks balanced salt solution, 0.1% bovine serum albumin (BSA), 20 mm HEPES [(4-(2-hydroxyethyl)-1-piperazineethanesulfonic acid)], pH 7.4. The cells were left to equilibrate at 28 °C for 30 min. The impedance measurement was performed on a CellKey instrument (MDS Analytical, Concord, ON, Canada). The signal was recorded for 5 min before the addition of the compounds, and 15 min thereafter. The cells in each well were stimulated once with a single concentration of compounds. The resulting data are expressed as the maximal signal corrected for the baseline and represented as a percentage of the full agonist effect (melatonin that serves as a positive control in these experiments). Naïve CHO-K1 cells were used as controls in these experiments as they do not show any signal after melatonin treatment.

## Figures and Tables

**Table 1:** Affinity of the compounds at human melatonin MT_1_ and MT_2_ receptors.

Compounds	Substituents	K_i_ MT_1_ (nM)	K_i_ MT_2_ (nM)
Melatonin	-	0.22 ± 0.01	0.33 ± 0.01
DIV4288	H	1300 ± 97	1.4 ± 0.1
DIV6518	F	630 ± 55	1.4 ± 0.3
DIV0879	Br	1779 ± 118	1.4 ± 0.1
DIV0880	I	1202 ± 143	1.0 ± 0.1
DIV6519	Ph	44 ± 7	0.49 ± 0.01
DIV6520	Sn(But)_3_	5000 ± 160	36 ± 3

Binding parameters of the compounds were obtained with 2-[^125^I]-melatonin as the radioligand. The data were obtained using membranes from CHO-K1 cells expressing either human melatonin receptors (hMT_1_ or hMT_2_) receptors. Results are given as the mean ± SEM for at least three experiments. SEM is standard error of the mean.

**Table 2:** Melatoninergic agonist potencies and efficacies on [^35^S]-GTPγS binding.

Compounds	Substituents	MT_1_		MT_2_	
		pEC_50_	E_max_	pEC_50_	E_max_
Melatonin	-	8.77 ± 0.66	100	9.20 ± 0.04	100
DIV4288	H	<5	ND	8.69 ± 0.37	34 ± 11
DIV6518	F	<5	ND	8.93 ± 0.66	30 ± 5
DIV0879	Br	<5	ND	8.86 ± 0.87	39 ± 9
DIV0880	I	<5	ND	9.48 ± 0.16	37 ± 5
DIV6519	Ph	<5	ND	<5	ND
DIV6520	Sn(But)_3_	<5	ND	<5	ND

Functional binding parameters of the compounds were obtained with [^35^S]-GTPγS as the radioligand. The data were obtained using membranes from CHO-K1 cells expressing either hMT_1_ or hMT_2_ receptors. Results are given as the mean ± SEM. for at least three experiments. ND: not defined. SEM is standard error of the mean. EC_50_ is defined as the half maximal effective concentration. It is sometimes expressed as its Log, pEC_50_. The Emax is the maximum possible effect for the agonist (melatonin).

**Table 3:** Potency and effect of the compounds on the cAMP production (inhibition).

Compounds	Substituents	cAMP MT_1_		cAMP MT_2_	
		pEC_50_	E_max_ (%)	pEC_50_	E_max_ (%)
Melatonin	-	9.46 ± 0.06	100	9.6 ± 0.12	100
DIV4288	H	<5	ND	8.74 ± 0.19	88 ± 6
DIV6518	F	<5	ND	8.55 ± 0.19	92 ± 14
DIV0879	Br	<5	ND	8.93 ± 0.19	100 ± 6
DIV0880	I	<5	ND	8.28 ± 0.22	90 ± 10
DIV6519	Ph	<5	ND	7.24 ± 0.56	143 ± 25
DIV6520	Sn(But)_3_	<5	ND	7.31 ± 0.10	94 ± 3

The data were obtained using CHO-K1 cells expressing either hMT_1_ or hMT_2_ receptors. Results are given as the mean ± SEM for at least three experiments. SEM is standard error of the mean. EC_50_ is defined as the half maximal effective concentration. It is sometimes expressed as its Log, pEC_50_. The Emax is the maximum possible effect for the agonist (melatonin).

**Table 4:** Potency and effect of the compounds on the β-arrestin recruitment.

Compounds	Substituents	β-Arrestin MT_1_		β-Arrestin MT_2_	
		pEC_50_	E_max_ (%)	pEC_50_	E_max_ (%)
Melatonin	-	9.49 ± 0.49	100	9.57 ± 0.06	100
DIV4288	H	5.65 ± 0.20	65 ± 1	7.65 ± 0.39	112 ± 16
DIV6518	F	5.66 ± 0.12	47 ± 0	7.00 ± 0.53	98 ± 20
DIV0879	Br	5.61 ± 0.48	65 ± 15	7.18 ± 0.59	141 ± 41
DIV0880	I	5.75 ± 0.25	41 ± 2	6.70 ± 0.57	101 ± 10
DIV6519	Ph	<5	ND	8.21 ± 0.76	16 ± 5
DIV6520	Sn(But)_3_	<5	ND	5.81 ± 0.49	25 ± 6

The data were obtained using cells expressing either hMT_1_ or hMT_2_ receptors. Results are given as the mean ± SEM for at least three experiments. SEM is standard error of the mean. EC_50_ is defined as the half maximal effective concentration. It is sometimes expressed as its Log, pEC_50_. The Emax is the maximum possible effect for the agonist (melatonin).

## Supplementary Materials

Supplementary materials can be found at www.mdpi.com/1422-0067/18/7/1347/s1.
