# Supplementary material for: New MT2 Melatonin Receptor-Selective Ligands: Agonists and Partial Agonists
Source: Int J Mol Sci. 2017 Jun 23;18(7):1347. doi: 10.3390/ijms18071347 (PMC5535840; doi:10.3390/ijms18071347)
Supplement: Supplementary file 1 [file ijms-18-01347-s001.zip › ijms-189445-supplementary-final.pdf]

Suplementary Materials

The characterizations of the molecules were done by mass spectrometry and NMR on the following instruments: 3100 series Waters mass spectrometer (Waters, Ma, USA) and a Bruker NMR AV300 instrument. The crude data are presented as figures in which the spectrum of the given compound is shown. All the mass spectra fitted the molecular masses expected for the compounds, and all the NMR spectra fitted the expected structures.

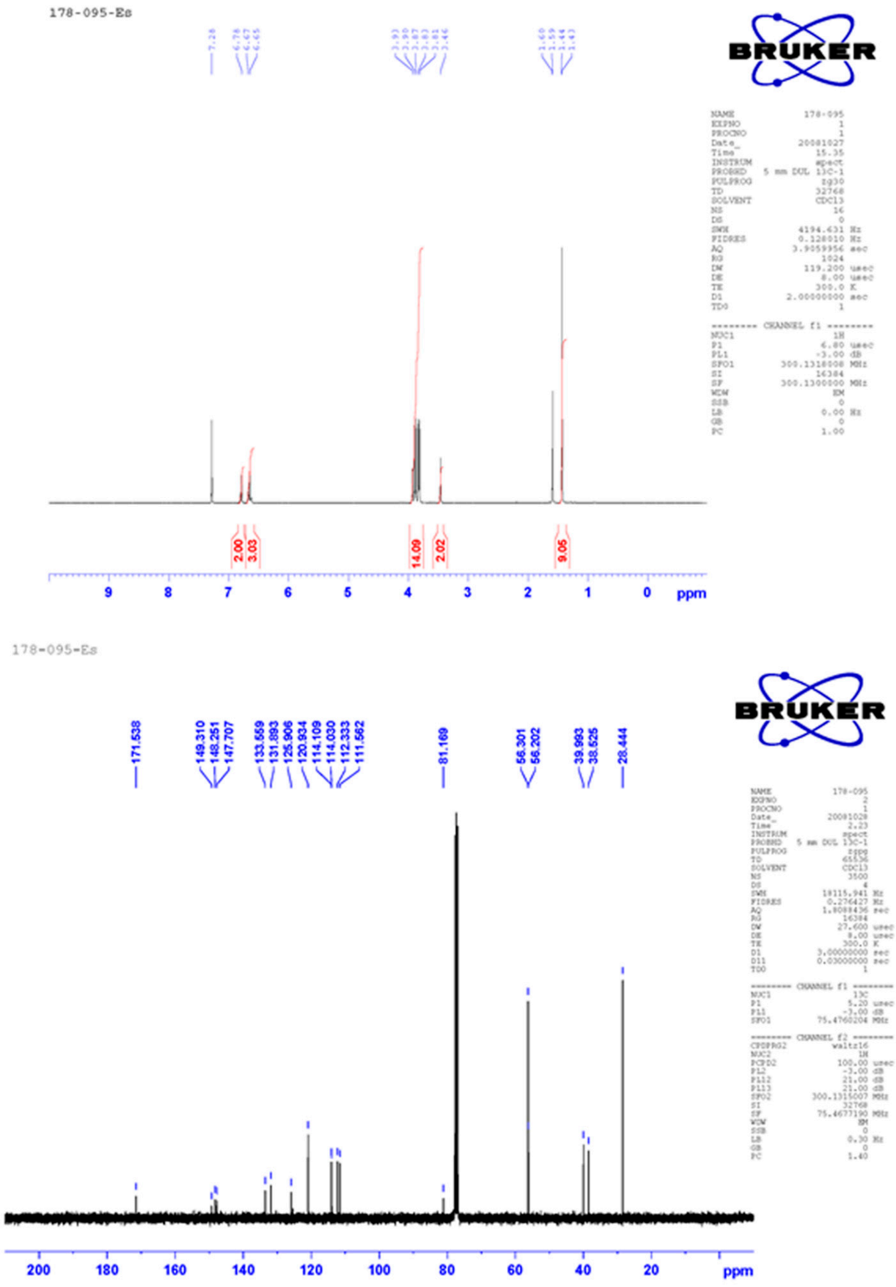

Supplementary Figure 1

Supplementary Figure 1: ^{1}H (top) and ^{13}C (bottom) NMR spectra of DIV4288

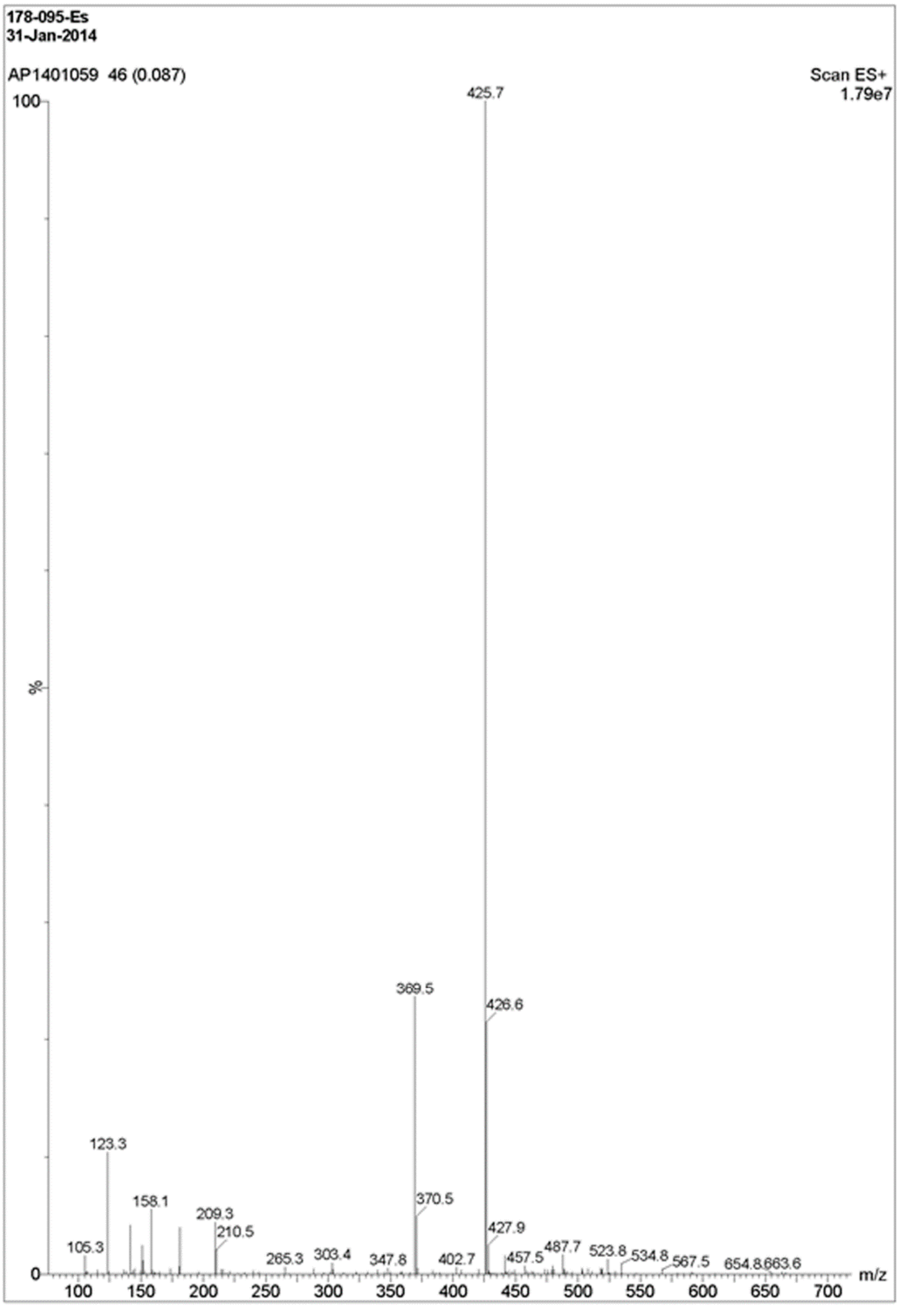

Supplementary Figure 2

Supplementary Figure 2: Mass spectrometry spectrum of DIV4288

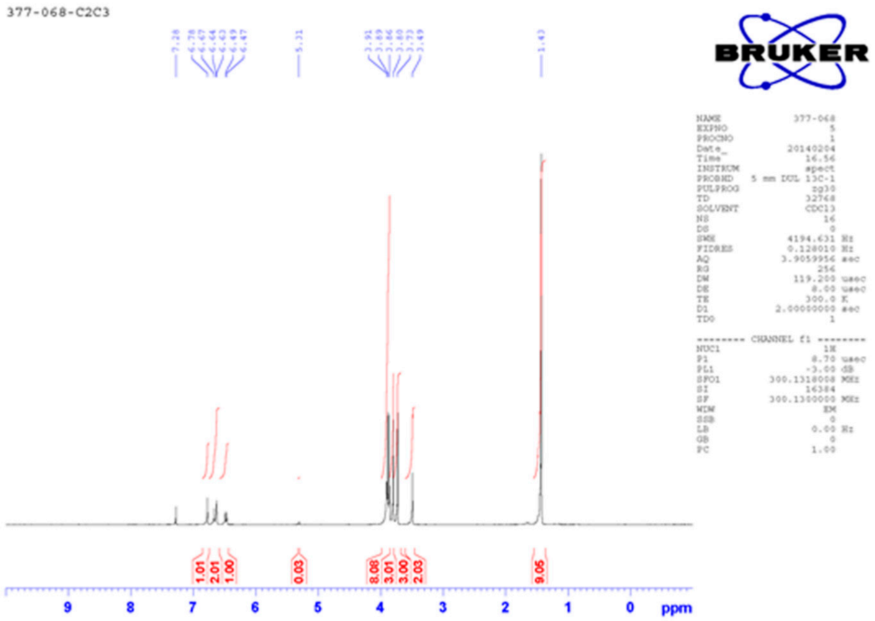

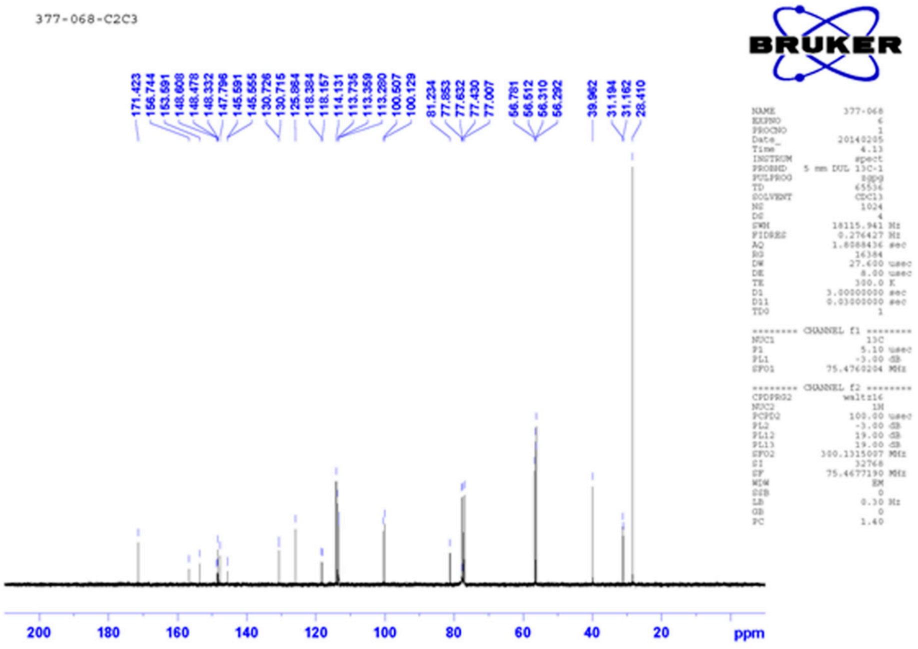

Supplementary Figure 3

Supplementary Figure 3: ^{1}H (top) and ^{13}C (bottom) NMR spectra of DIV6518

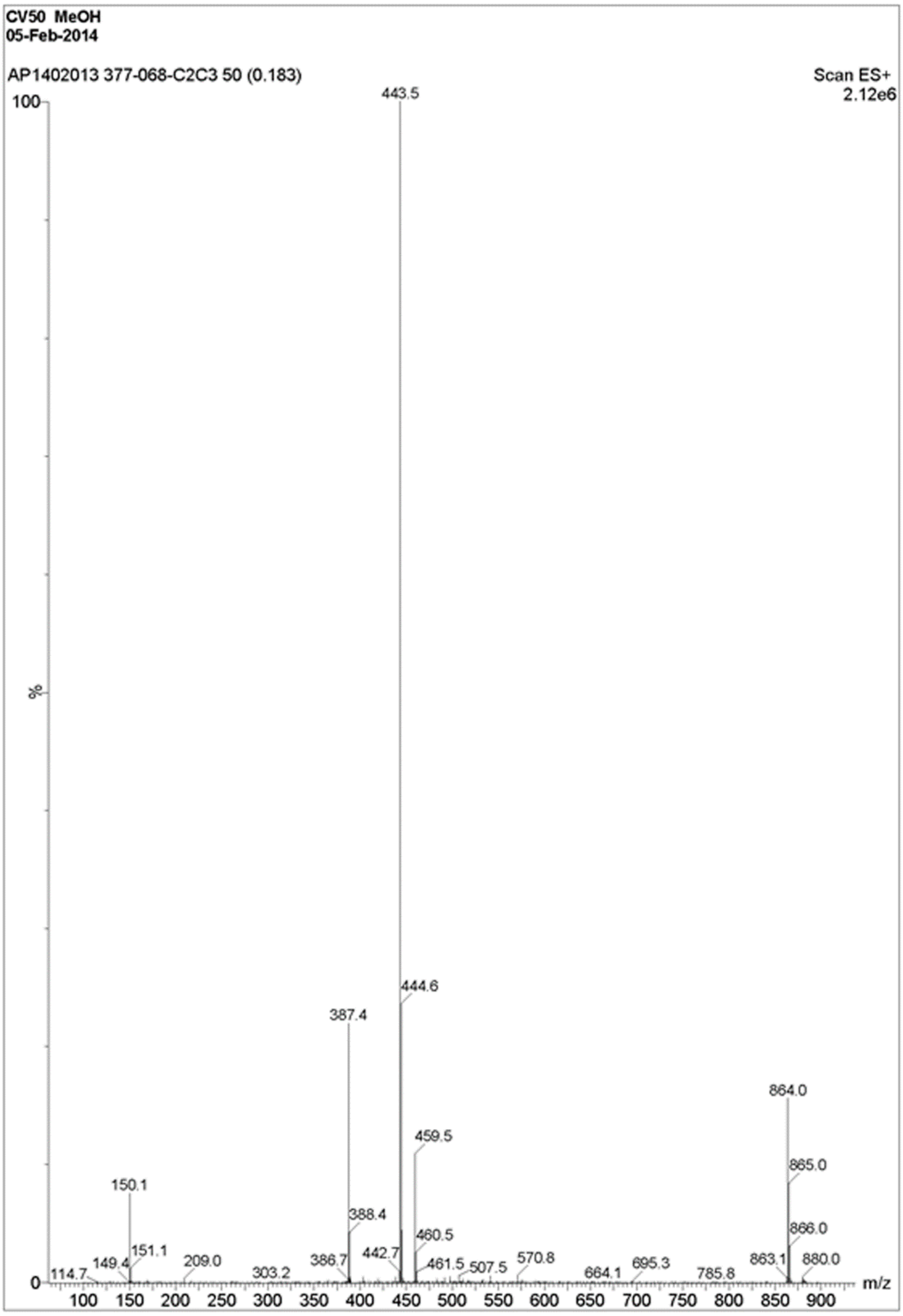

Supplementary Figure 4

Supplementary Figure 4: Mass spectrometry spectrum of DIV6518

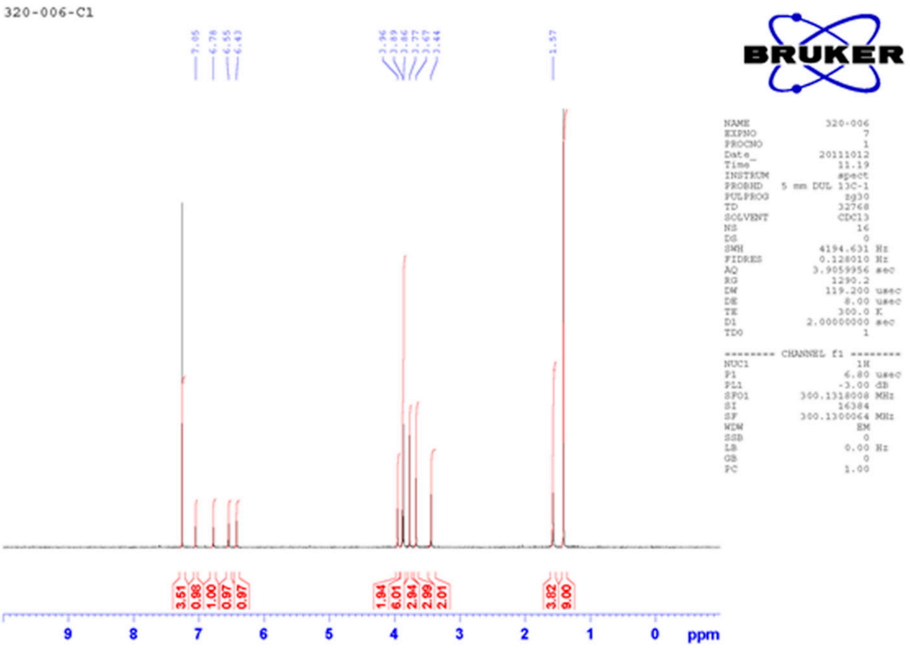

Supplementary Figure 5

Supplementary Figure 5: ^{1}H NMR spectra of DIV0879

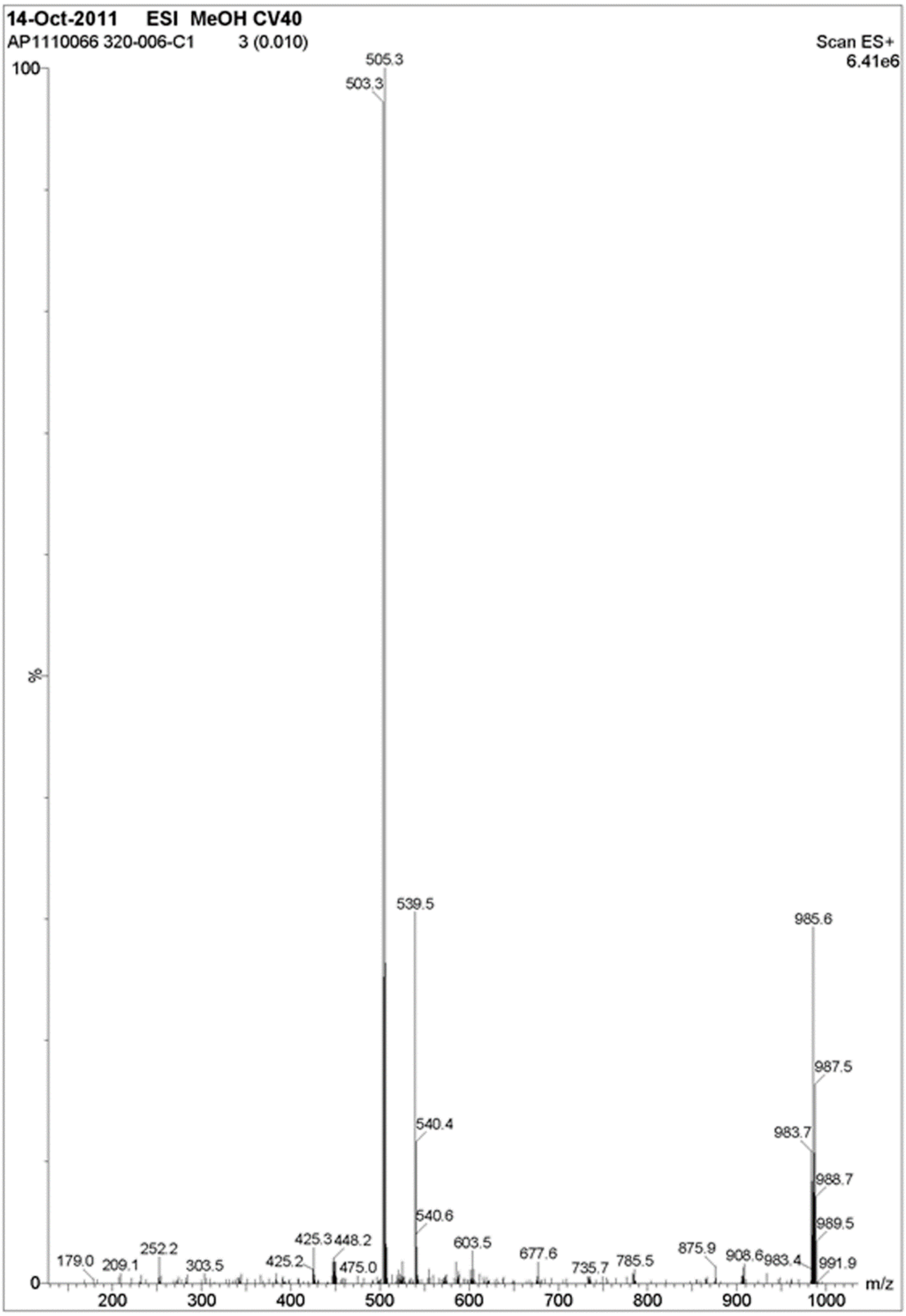

Supplementary Figure 6

Supplementary Figure 6: Mass spectrometry spectrum of DIV0879

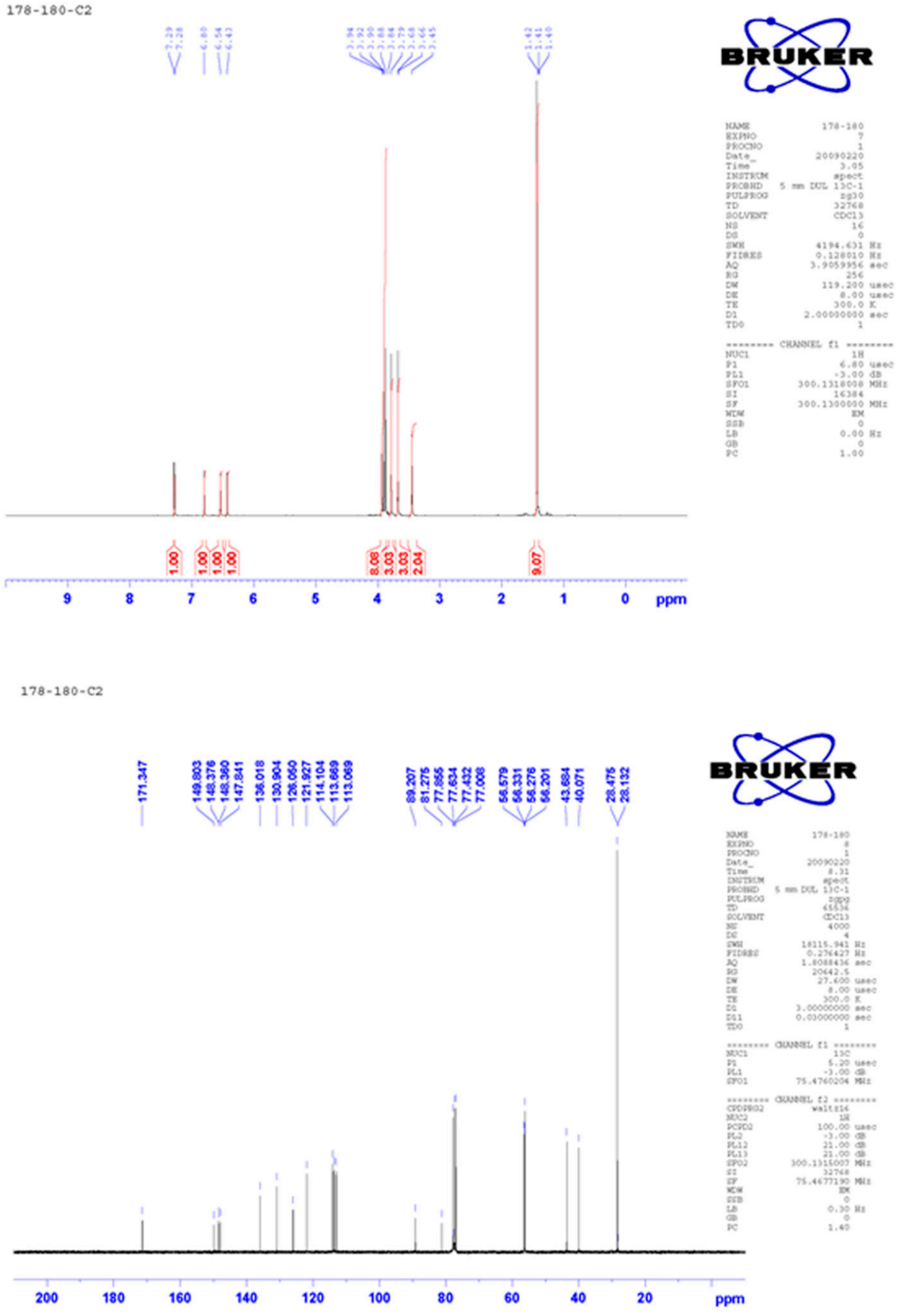

Supplementary Figure 7

Supplementary Figure 7: ^{1}H (top) and ^{13}C (bottom) NMR spectra of DIV0880

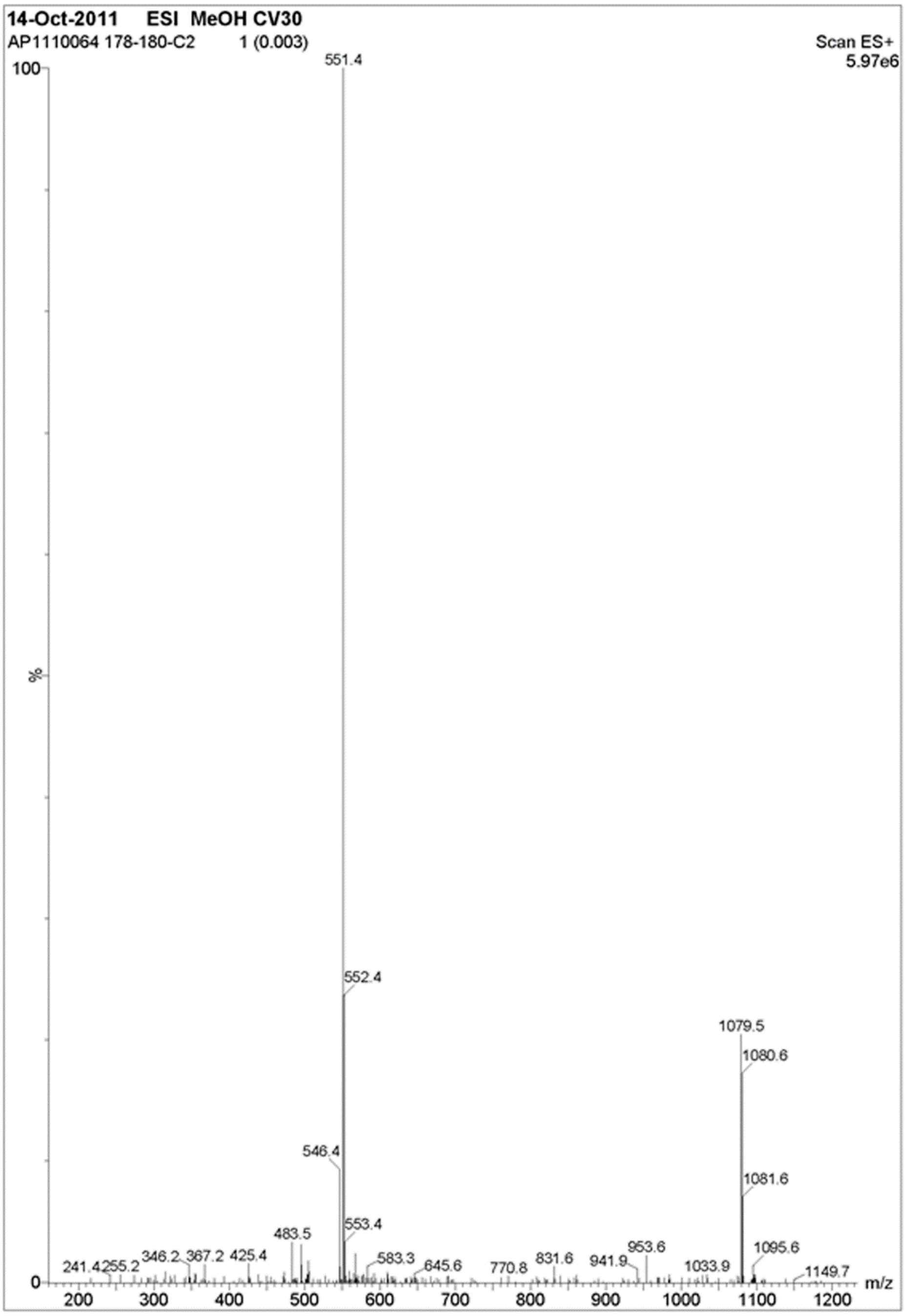

Supplementary Figure 8

Supplementary Figure 8: Mass spectrometry spectrum of DIV0880

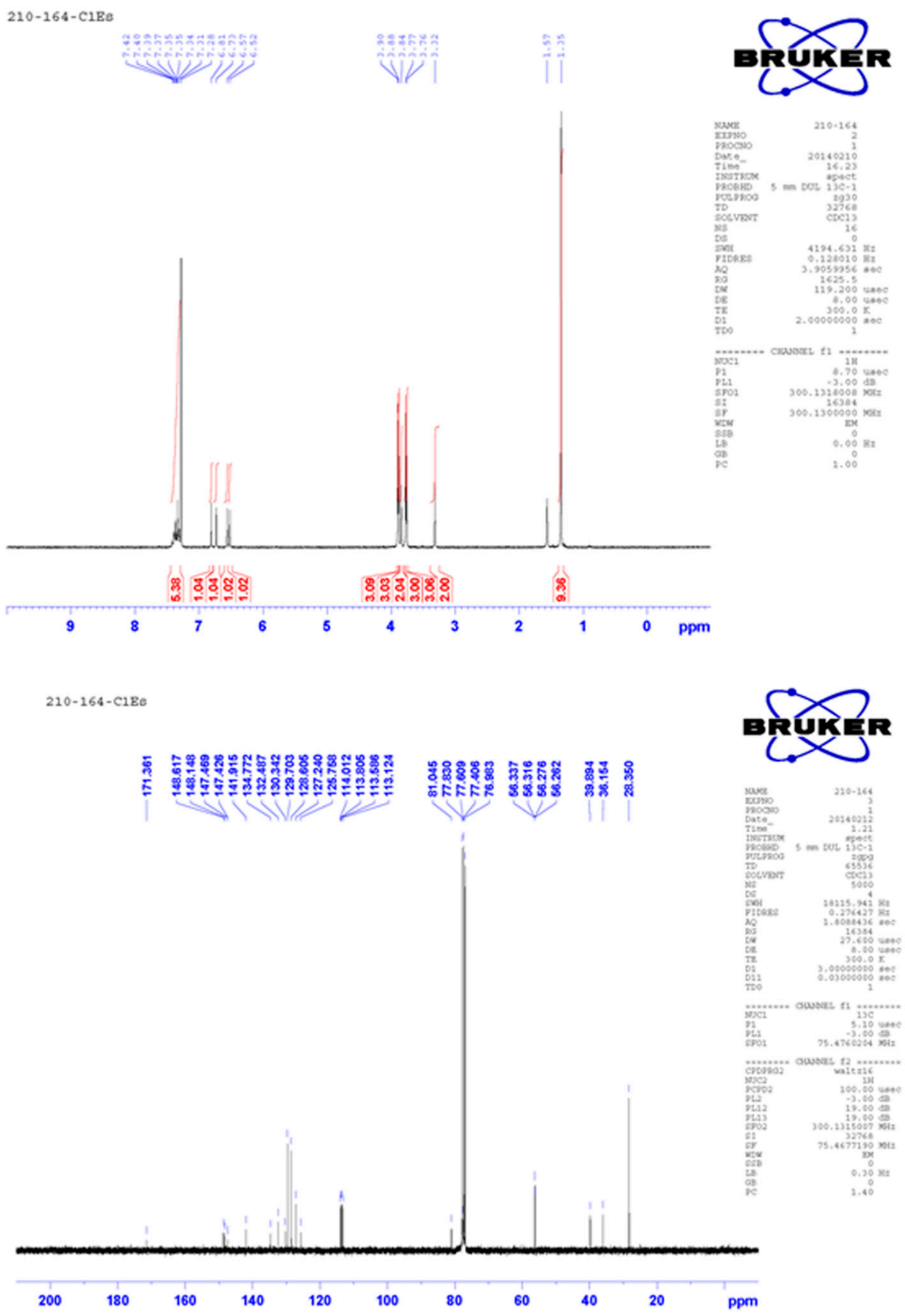

Supplementary Figure 9

Supplementary Figure 9: ^{1}H (top) and ^{13}C (bottom) NMR spectra of DIV6519

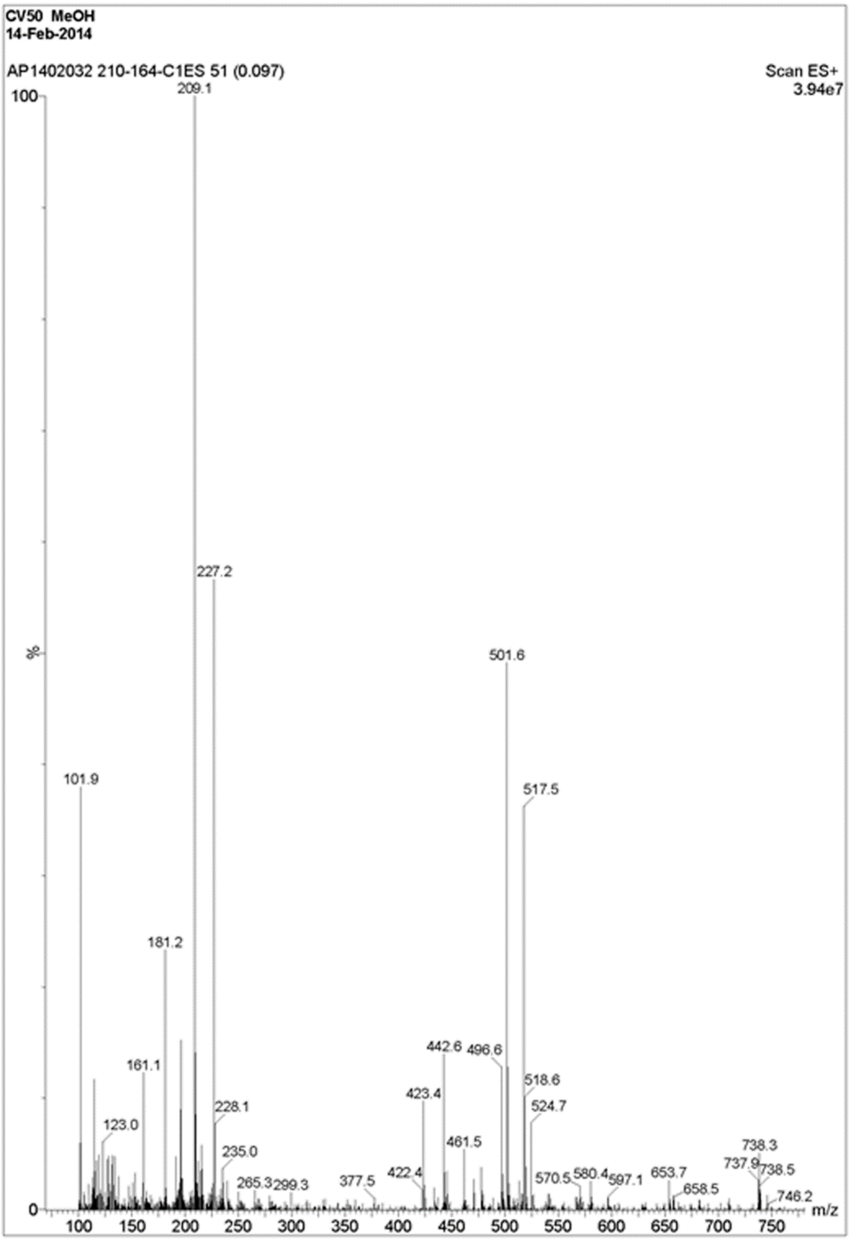

Supplementary Figure 10

Supplementary Figure 10: Mass spectrometry spectrum of DIV6519

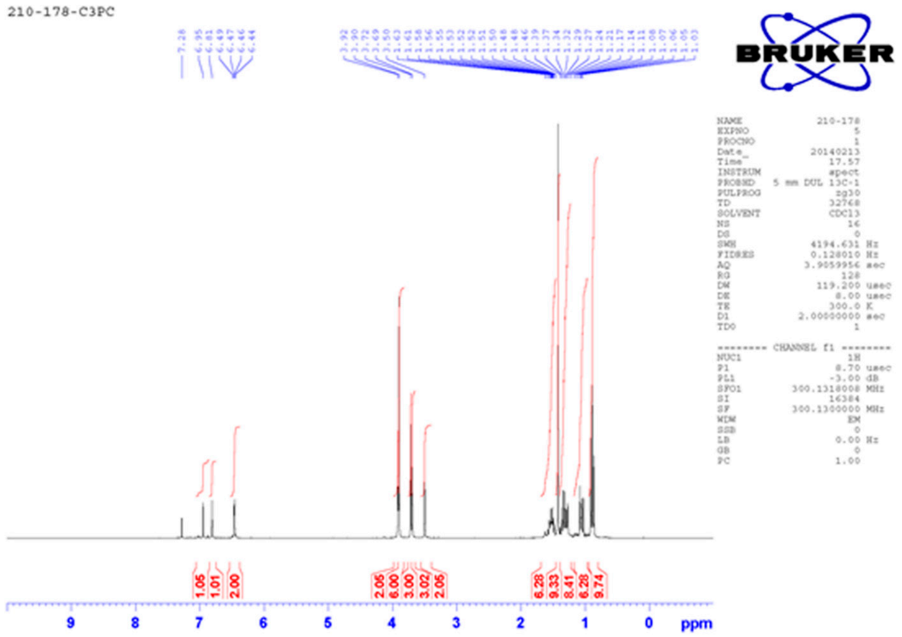

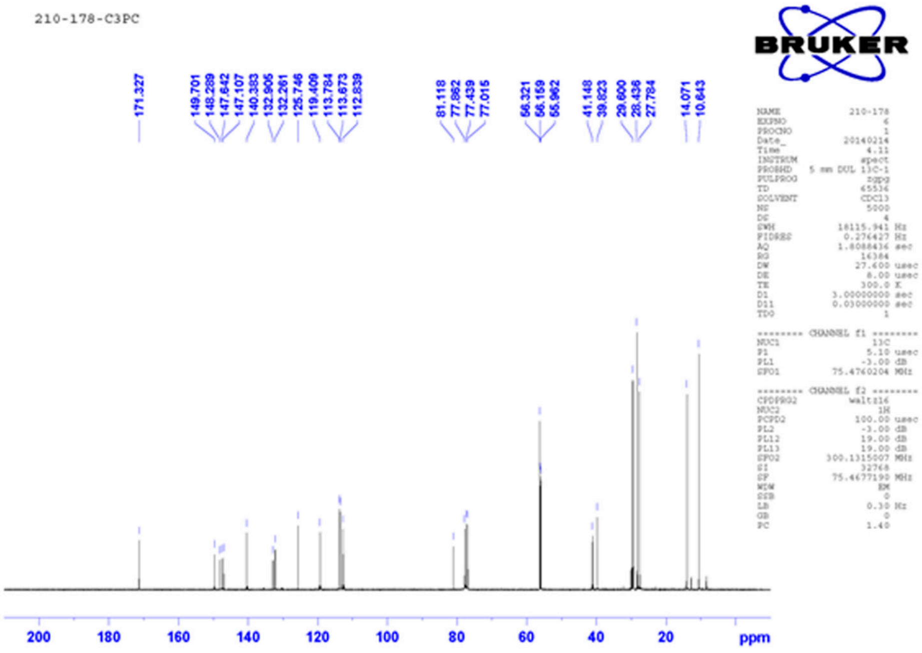

Supplementary Figure 11

Supplementary Figure 11: ^{1}H (top) and ^{13}C (bottom) NMR spectra of DIV6520
